# The Neural Correlates of Narcissism: Is There a Connection with Desire for Fame and Celebrity Worship?

**DOI:** 10.3390/brainsci13101499

**Published:** 2023-10-23

**Authors:** Sydney Ash, Dara Greenwood, Julian Paul Keenan

**Affiliations:** 1 Cognitive Neuroimaging Laboratory, Montclair State University, 320 Science Hall, Montclair, NJ 07043, USA; 2 Department of Psychology, Vassar College, Poughkeepsie, NY 12604, USA

**Keywords:** fame, celebrity worship, narcissism, frontal cortex, frontostriatal

## Abstract

(1) Objective: Narcissism is characterized by emotional regulation deficits, a lack of empathy for others, and extreme self-focus. Narcissism has also been linked to an increased desire for fame and celebrity worship. Here, the neuroscience underlying narcissism is examined in order to determine what regions and networks of the brain are altered when non-narcissistic individuals are compared to participants with both grandiose and vulnerable narcissism. (2) Methods: The behavioral relationships between grandiose narcissism and desire for fame and vulnerable narcissism and celebrity worship are explored, along with a possible relationship at the neural level between these constructs. In this paper, we review research demonstrating that increased levels of grandiose narcissism are associated with an increase in obsession with fame, while vulnerable narcissism is associated with celebrity worship. (3) Results: Based on current data, the frontal regions underlie narcissism and also likely underlie celebrity worship and desire for fame. This tenuous conclusion is based on a limited number of studies. (4) Conclusions: The brain areas associated with grandiose narcissism may be associated with an intense desire for fame as well, while brain regions associated with vulnerable narcissism may be similar in celebrity worshipers. Future research studies on the brain that are specifically designed to test these relationships at a neurological level are needed.

## 1. Introduction

Narcissism has been broadly defined as a relatively stable personality trait consisting of grandiosity, excessive self-focus, and a lack of empathy [1]. Narcissism can present as Narcissistic Personality Disorder (defined by the DSM) or as more normative/subclinical narcissistic tendencies, defined by high scores on measures such as the Narcissistic Personality Inventory (NPI; Raskin & Terry, 1988 [2]) or Single-Item Narcissism Scale [3]. The extent to which an individual may fall into clinical vs. subclinical narcissistic tendencies may be conceptualized as a matter of degree and disruption; those with NPD would score highly on more normative measures but would also demonstrate marked interference with life functioning and more consistent or extreme thoughts and behaviors. Research on the neural correlates of narcissism focus largely on subclinical grandiose narcissism using the NPI [3,4,5,6,7,8], while a few studies have used fMRI to examine the brains of patients with NPD [9,10]. Some studies have also investigated NPD by employing the use of DTI in both volumetric and resting state [11,12].

Beyond considerations of narcissism as a set of individual characteristics, it is important to place the phenomenon in a cultural context. For example, scholars have theorized that today’s media-saturated society, combined with increased economic anxiety and competition for jobs, may partly explain the apparent inflated self-focus and decreased empathy of younger generations [13]. Perhaps not coincidentally, scholars have also noted a rise in the appeal of fame or celebrity culture in recent decades [14], a trend that may be fueled by the ubiquity of social media platforms that not only enable increased access to celebrities but also provide opportunities for anyone with a phone or laptop to cultivate their own “brand” or mass following online. Indeed, research has shown a link between the desire for fame and increased engagement with celebrities on social media [15]. Finally, there is a substantial number of works linking increased narcissism to increased fame interest and celebrity affinities [16,17,18,19]. Ultimately, we propose that similar neural correlates will emerge when examined in the context of fame interest and, possibly, parasocial engagement (i.e., perceived socioemotional connection, [20]) with celebrities. This paper will review the developmental context of both narcissism and fame appeal and their relationship with each other before focusing on the research on the neural correlates of narcissism. Searches were initiated in PubMed, PsychInfo, Google Scholar, and Scopus. Our initial search terms included ‘brain’, ‘fMRI/MRI’, ‘TMS/rTMS’, ‘neural’, ‘fame’, ‘celebrity’, ‘desire for fame’. We also keyed in on the current authors’ relevant work as well as that of known authors in the field (e.g., ‘McCutcheon’). From here, articles were selected and categorized into the following headings (see below).

## 2. Measures of Narcissism, Desire for Fame, and Celebrity Worship

Celebrity worship, as defined by McCutcheon, Lange, and Houran (2002), is an intense form of celebrity attachment that is oftentimes represented by a spectrum that starts at ‘passionate fan’ and ends at borderline pathological [21,22]. The Celebrity Attitude Scale (CAS) is often used when determining the intensity of a fan’s celebrity worship behavior. The CAS was developed to “identify individuals who are overly absorbed or addicted to their interest in a celebrity” [22].

Desire for fame is defined as “a desire to be seen and appreciated by as many people as possible” [17], and has been measured by Greenwood et al.’s (2013) Fame Appeal Scale (FAS). The scale consists of three components of desire for fame: visibility (i.e., desire to be recognized in public), status (i.e., desire for a wealthy lifestyle), and prosocial (i.e., using fame to help others) [18].

It may also be of benefit to mention that the Narcissistic Personality Inventory (NPI) is most often used to assess for traits of grandiose narcissism, while the Maladaptive Covert Narcissism Scale (MCNS) is used to determine the presence of vulnerable narcissism. There are multiple versions of these scales, with each differing in the number of items included and answer format (e.g., a forced choice between two items or a Likert scale that goes from Strongly disagree to Strongly agree).

## 3. Development of Narcissism, Desire for Fame, and Celebrity Worship

It has been suggested that narcissism in children originates when parents overevaluate them, thinking that their child is special and more entitled than others [23]. Through social learning theory, these children internalize their parents’ views, giving them an unnaturally high self-esteem and resulting in personality traits associated with narcissism [23].

Levels of narcissism amongst U.S. college students have increased over the past 25 years, and students with narcissistic traits often display a sense of entitlement when in academic settings [24]. The cultural transformation towards more individualistic values in Western societies, compared to the former collectivistic cultures, has been blamed for the recent increase in narcissism [25]. In an individualistic culture, people stress the importance of independence and self-sufficiency, while in collectivistic cultures, people often put the group first and individuals second [26]. Due to this shift in values, it has been proposed that an increased focus on the accomplishments of the self has led to an increase in the occurrence of narcissistic traits. In the classroom, these narcissistic individuals are often rewarded for their behavior due to their higher self-esteem, extraversion, and participation and leadership skills [27]. Due to higher levels of praise, students often continue to express these characteristics, some of which may not end up benefiting them in a proper work environment. 

A concept similar to narcissism, hubris, is a personality characteristic that includes exaggerated pride and overwhelming self-confidence [28]. A study conducted on persons in leadership positions (gerontological nurses during the SARS-CoV-2 pandemic) attempted to determine if there were emotional correlates related to experienced hubris in leadership positions. The results of this study indicated that the report of more negative affective states, such as being fearful, sleepy, and sad, correlated with less hubristic symptomatology [28]. As narcissism has the possibility to be developed from the time an individual is born, hubris often occurs after one gains a position of power. Gender was also found to have no effect on the differentiation of responses, which is oftentimes the opposite in terms of narcissism.

It is of note that gender differences often play a role in the expression of narcissistic personality traits. One study revealed that there was a consistent gender difference in narcissism, with men scoring a quarter of a standard deviation higher in narcissism than women [29]. This result is not surprising, as men have also displayed differences in other personality traits, such as risk-taking behaviors [29,30], occurrence of neuroticism [29,31], and self-esteem [29,32]. A study conducted by Yang et al., (2015) revealed that these differences in narcissistic personality between genders may be due to brain structure. Through the use of VBM, they determined that the relationship between narcissism and gray matter volume varied based on gender [33]. Resting state functional connectivity (rsFC) was also examined, and it was found that the NPI scores of females were negatively correlated with rsFC strength between the right superior parietal lobe and the right precuneus [33]. However, the opposite relationship was found in males [33]. The differences in narcissistic personality traits seem to be correlated with the variability in the structure and function of the brain based on gender. 

In terms of desire for fame and celebrity worship, some studies have revealed that females are more prone to celebrity worship [34,35], while other studies have concluded that there are no gender differences regarding this phenomenon [16]. One study that examined celebrity worship along with belongingness needs discovered that females had slightly higher scores in both celebrity worship and the need to belong in comparison to males [35]. Once again, this indicates the existence of slight gender differences that should be taken into account when conducting research on narcissism, desire for fame, and celebrity worship. 

## 4. Relationship between Narcissism and Desire for Fame

It has been observed that subjects with narcissistic traits tend to focus on the recognition and elite status that fame provides and that future fame is a more likely outcome for narcissistic individuals in comparison to less narcissistic individuals [18]. Numerous studies have found a correlation between narcissism and the intense desire to be famous, indicating that there is an overlap between higher FAS scores and higher NPI scores [16,17,18,19].

Another aspect of narcissism that is strongly associated with desire for fame is fantasy proneness and the use of fantasy to cope with stress [36]. A narcissist is often portrayed as someone whose inner experience is overwhelmed by grandiose images of themselves and idealized others [36]; they live in a world of fantasy where their mind is able to manipulate both their own personality and the personalities of the people around them. One study on narcissistic individuals found that they “show an inclination to experience achievement, heroic, sexual, hostile, self-revelation, and future-oriented daydreams” [36]. Not only do they daydream more often, but they are known to be more willing to believe those daydreams as well. 

Factors such as fantasy proneness, unstable self-esteem, and issues with self-image are all connected to an individual’s self-awareness, a phenomenon of self-knowledge that is central to consciousness [37,38]. The neural correlates of self-awareness have been determined to be rooted in the medial prefrontal cortex (mPFC), as the mPFC is involved in self-reflective thought, projection of the self into the future, and self-regulation in social settings [37]. Studies have determined that the right dorsolateral prefrontal cortex and bilateral medial prefrontal areas, which are related to a lack of control over emotional and social interactions, may also play a role [5,8,9,12,39,40]. Even though concrete evidence linking desire for fame to these brain areas has not yet been obtained, the relationships between narcissism, desire for fame, and self-awareness seem to point to the frontal regions of the brain as possible neural correlates.

## 5. Relationship between Narcissism and Celebrity Worship

A 2018 study exploring the relationship between narcissism, fame appeal, and attitudes toward celebrities found that the appeal of fame is associated with attachment to celebrities [17]. It was found that overall fame appeal was associated with grandiose narcissism (characterized by a need for attention and dominance and a sense of superiority), while positive celebrity attitudes were more strongly predicted by vulnerable narcissism (characterized by distrust, anxiety, and hostility towards others) [17].

In another study conducted by Ashe, Maltby, and McCutcheon, it was found that problematic dimensions of celebrity worship, such as relationships in the borderline-pathological subscale, were positively associated with narcissism [16]. The reasoning behind this correlation could be that narcissists are able to convince themselves of their amazing social skills, ultimately putting themselves on the same level as other celebrities.

Even though extensive research has not yet been conducted on the neural correlates of celebrity worship, similarities in the traits of narcissism, desire for fame, and celebrity worship, such as fantasy proneness [17,36,41] and self-esteem issues [11,42,43], indicate a possible relationship with the prefrontal regions. It is reasonable to hypothesize that the cause of these deviations of the self may lie within brain areas such as the mPFC, but further research on the brain is needed to confirm the neural correlates.

## 6. Differentiating Grandiose and Vulnerable Narcissism in Research

Numerous studies indicate that there are two distinct types of narcissism: grandiose and vulnerable narcissism [17,44,45]. As defined by Abeyeta, Routledge, and Sedikides (2017), grandiose narcissism is typically “characterized by a perception of self-importance and social power” [17,46], while vulnerable narcissists share the same sense of entitlement but also experience “lower self-esteem and greater anxiety and depression” [17].

It has been determined that narcissism, desire for fame, and celebrity worship are related on a behavioral level [16,17,18,19]; however, here, we attempt to determine if they could be related on a neural level as well. These three deviations of the self have been associated with the frontal regions of the brain, including the medial prefrontal cortex [5,6,10,12,39] and the right dorsolateral prefrontal cortex [5,8,9,12,39]. Even though neurological studies are in their infancy, the relevant data suggest a common neural pathway that involves the interplay of empathy [6,10,47] and self-awareness [9,10,37,45] in the frontal regions of the brain.

In a study conducted by Zhang et al. in 2015, it was concluded that overt, or grandiose, narcissism was negatively correlated with emotion regulation difficulties, while covert, or vulnerable, narcissism was positively correlated with this factor [45]. In order to reach this conclusion, the researchers used respiratory sinus arrhythmia (RSA) reactivity and the NPI, HSNS, and difficulties in emotion regulation scale (DERS) in 227 students to determine the relationship between narcissism and emotion regulation. RSA is known to “reflect the variation in the interbeat intervals (IBIs) of the heart at the frequency of breathing” and has been associated with physiological emotion regulation [45]. Studies have shown that high resting RSA is related to better emotional regulation, while low resting RSA is related to negative emotionality and behavior problems [45]. Higher RSA reactivity, or a decrease in RSA due to a stress-inducing task, is also related to a higher ability to control emotions [45]. Grandiose narcissism showed significant negative correlations with lack of emotional awareness and clarity, whereas vulnerable narcissism showed significant positive correlations with overall emotion regulation difficulties, impulse control difficulties, and lack of emotional clarity [45]. While grandiose narcissism was not determined to be significantly associated with an RSA decrease or trouble regulating emotions, individuals with vulnerable narcissism and high levels of RSA decrease were associated with emotion regulation difficulties. It was concluded that vulnerable narcissists require higher RSA reactivity because it provides a buffer from suffering greater problems with emotion regulation [45]. These results suggest that the types of narcissism are completely separate in terms of their impact on mental health, emotional dysregulation, and emotional reactivity, with vulnerable narcissism being more maladaptive in terms of regulating emotions [45].

In a review by Förster and Kanske (2022), it was hypothesized that compassion has the ability to act as an efficient form of positive emotion regulation. Areas of the brain such as the ventral striatum, anterior cingulate cortex, and orbitofrontal cortex are responsible for the production of strong positive emotions such as self-compassion [48]. Individuals with higher levels of vulnerable narcissism specifically tend to experience disparities in intrapersonal characteristics and interpersonal desire for acceptance, resulting in an inflation of the self to cover their vulnerabilities [48]. Vulnerable narcissists may benefit from self-compassion, as it broadens their scope of action, enabling them to find coping strategies other than negative emotion regulation.

The above studies suggest that there are differences in the personality traits and characteristics of individuals with grandiose narcissism and vulnerable narcissism. In terms of desire for fame and celebrity worship, it may be possible that individuals with grandiose narcissism view the appeal of fame and celebrity culture in a different light compared to individuals with vulnerable narcissism.

## 7. Narcissism and the Brain

### 7.1. Cortical Thickness and Volume

Researchers have employed 3D-T1W MRI scanning in an attempt to discover unique processing and anatomy in various brain regions in people with narcissism. Such studies have provided evidence of differences such as reduced frontal cortex thickness and cortical volume [9] and weakened frontostriatal connectivity [11] when comparing these areas to the same brain regions of people who scored lower on scales of narcissism. These studies (and those detailed below) postulate that differences in cortical thickness and volume, gray matter deficits, and frontostriatal connectivity in certain regions of the brain, including the frontal cortex, may be related to negative effects such as impairment of social cognition and an impaired ability to regulate emotions [9], along with a lack of empathy [6,10,47] and increased anger and aggression [6,44].

Another study used a sample of 183 healthy college and postgraduate students in order to determine differences in cortical thickness (CT) and cortical volume (CV) in people with traits of pathological narcissism. The study’s subjects were given the Pathological Narcissism Inventory (PNI), and MRI scanning and surface-based morphometry was used in order to calculate the CT and CV. It was found that a higher score on the PNI was associated with “decreased CV of the left medial prefrontal cortex and the right postcentral gyrus as well as decreased CT of the right inferior frontal cortex” [9]. It was also found that “decreased CT and CV in the social brain network among participants with higher PNI score might be consequently related to impairments in social cognition”, such as empathizing with others, regulating emotions, and controlling behavior [9]. They also found decreased CT and CV in the dorsolateral prefrontal cortex, which is a brain region highly associated with emotion regulation [9]. Specifically, decreased CT and CV in this area would result in a limited ability to recognize and regulate their thoughts and feelings, “implying that they are also less capable of recognizing their own emotions and the underlying causes” [9]. Due to this disconnect between the self and self-awareness, individuals may have a harder time regulating negative emotions because they are not aware of the cause of these feelings. Other studies have shown that reductions in cortical thickness in these brain regions also result in extreme sensitivity to feedback from others, especially negative feedback that may provoke extreme emotion [9]. This would explain why people with narcissistic personalities crave positive feedback and attention; grandiose narcissists desire the attention that celebrities receive and develop an intense desire to become famous themselves. Vulnerable narcissists also require reassurance in order to soothe insecurities but find social situations more stressful, resulting in less of a desire for fame.

### 7.2. Gray Matter Deficits

Narcissism has also been correlated with a lack of control over emotions during social interactions, which may be related to issues with self-awareness. Another study examined six male patients with narcissistic personality disorder. In this study, T1W-MRI revealed prefrontal gray matter deficits and potential alterations in prefrontal white matter [12]. Specifically, the gray matter deficits were found in the right prefrontal and bilateral medial prefrontal areas, which are related to a lack of control over emotional and social interactions. One of the clusters was located in the medial prefrontal cortex, an area that has been identified as having a strong correlation with the self and self-awareness [9,10,37,45]. A lack of self-awareness in narcissistic individuals may lead to issues with self-reflective thought [37] and self-regulation in social settings [9,37,45]. Considering that this area is often associated with narcissism, it may also be a critical region to study when looking at the parts of the brain linked to intense desire for fame. These results were replicated in another fMRI study, which revealed that high narcissistic traits in a sample of healthy subjects were “associated with diminished activation in the right anterior insula, but also right dorsolateral prefrontal cortex and lateral premotor cortex” [5].

The interplay of emotional empathy [6,10,47] and self-awareness [9,10,37,45] in the dorsolateral and medial prefrontal cortex seems to play a major role in the presence of narcissism. In another structural MRI study involving gray matter (GM) deficits, researchers studied 17 patients that had been diagnosed with narcissistic personality disorder. When comparing the NPD patients to healthy controls, the patients had significantly smaller gray matter volumes in the left anterior insular region, the bilateral superior frontal gyrus (including the dorsolateral and medial areas), and the bilateral middle frontal gyrus [10]. Smaller GM volumes were also found in the rostral and median cingulate cortex, as well as the dorsolateral and medial parts of the prefrontal cortex [10]. These brain regions are known to be associated with empathic tendencies, and a positive correlation was found between emotional empathy and GM volume in the left anterior insula [10]. Neuroimaging studies have discussed “the involvement of the anterior insula, medial prefrontal cortex and anterior cingulate cortex during self-referential processing and decision-making” [10], and it could be argued that these differences in gray matter volume also affect the egocentric perspective of patients with narcissistic personality disorder due to a decrease in GM volume. This decrease in volume negatively impacts the self-awareness of an individual, as their self-referential processing and ability to make decisions for themselves suffers. These effects, coupled with a lack of emotional empathy for others, cause a disconnect between a narcissist and the outside world, often resulting in self-obsession. Again, the differences in specific brain regions have led researchers to believe that narcissism creates a personality type often characterized by self-focus, causing them to crave external attention and validation and therefore increasing their desire for fame.

### 7.3. Frontostriatal Connectivity 

The medial prefrontal cortex (mPFC) has been associated with the occurrence of narcissism in multiple studies [5,6,10,12,39]. The mPFC is largely related to the concept of self-awareness and has been correlated with self-referential processing [10,37], self-reflective thought [37], and self-regulation in social settings [9,37,45]. One study determined that prefrontal structural deficits, or reduced mass in the frontal region, resulted in the development of NPD [12], while another study concluded that weakened connectivity amongst frontal regions, including the mPFC, plays a role [11]. Since the mPFC has been determined to be an important neural correlate in the occurrence of narcissism, issues with self-awareness have also been identified as a narcissistic personality trait.

An additional study employed diffusion tensor imaging in order to assess the frontostriatal connectivity of 50 healthy undergraduate students, along with testing them for traits of grandiose narcissism. The researchers concluded that white matter integrity in the frontostriatal pathway was negatively correlated with narcissism, which causes a disconnect between the self and the reward system of the brain [11]. It is thought that due to this lack of connectivity between the self and rewards, grandiose narcissists may seek external validation in order to compensate for disparities between baseline and desired self-rewards [11]. Another theory suggests that narcissists require a larger amount of affirmation from others in order to reach their desired levels of self-esteem [11]. Both of these explanations illustrate the strong correlation between grandiose narcissism and desire for fame, considering the amount of external validation that is necessary for narcissists to obtain a normal level of self-esteem. It would also be reasonable to predict that connectivity in the frontostriatal pathway of a person with intense desire for fame would be similar to the connectivity in the brain of a narcissist observed in this study. In terms of vulnerable narcissism, vulnerable narcissists are also tethered to external validation because of their highly negative view of self, but the stress of social situations results in more celebrity worship and a decreased desire for fame [17].

In a study of 168 undergraduate and graduate students, the participants were given the Narcissistic Personality Inventory (NPI) in order to test for grandiose narcissism before undergoing a five-minute rs-fMRI scan. The researchers were able to determine that grandiose narcissism was associated with “resting-state functional connectivity (RSFC) across multiple neural systems, including functional connectivity between and within limbic and prefrontal systems as well as their connectivity with other networks” [6]. RSFC is a task-independent neuroimaging measure that allows for the examination of neural systems that may be associated with narcissism [6]. The RSFC patterns within each individual are unique; this gives researchers the opportunity to discover the patterns of connectivity associated with differences in specific personality traits or cognitive functions such as narcissism [6]. The key areas that were found to be linked to narcissism were the amygdala, the lateral and mPFC, and the anterior cingulate regions. It was found that the connections between these regions were primary predictors of the NPI scores, with higher positive network strength resulting in a higher score on the NPI [6]. These brain regions are associated with many characteristics of narcissism, such as emotion regulation deficits, exaggerated anger during negative social interactions, lack of empathy for others, and self-occupation [6]. This excessive self-focus implies a strong correlation between narcissism and an extreme desire for fame, as narcissists are known to have a “preoccupation with fantasies of unlimited success, power, beauty, and similar values” [49]. The researchers in this study also concluded that the development of grandiose narcissism is most likely rooted in interactions across multiple neural systems instead of individual regions of the brain [6].

### 7.4. Reaction Time through TMS

Self-awareness and recognition in the prefrontal cortex also seem to be impacted when comparing individuals with subclinical grandiose narcissism to healthy individuals. In a study involving transcranial magnetic stimulation, 11 healthy participants were used in order to determine the neural correlates of self-recognition in different regions of the right prefrontal cortex (PFC) and whether reaction time was related to subclinical grandiose narcissism [8]. After participants took the NPI, they reacted to a series of faces, including images of themselves, same-sex familiar faces, and same-sex stranger faces. It was found that reaction time when looking at images of the self was positively correlated with narcissistic personality traits, and increases in the expression of narcissistic traits were associated with a decrease in reaction time during self-recognition in comparison to the recognition of familiar faces and the faces of strangers [8]. In other words, patients scoring higher on the NPI were able to recognize their own faces faster than other individuals scoring lower on the NPI [8]. It was concluded that the results may suggest a trend toward greater physical self-awareness [8,50] in the participants that expressed narcissistic personality traits. This indicates that the PFC may also be a neural correlate of grandiose narcissism, as inflated self-focus allows for a quicker reaction time when recognizing oneself [8]. Even though this study did not include research on vulnerable narcissism, it is important to note that vulnerable narcissists are also highly self-focused; thus, they may have provided similar results if their reaction time was studied.

### 7.5. Psychopathy and NPD

Psychopathy and narcissism have been determined to be overlapping constructs as both express similar characteristics, such as a tendency for grandiosity, impulsivity, and limited empathy [51]. Through the use of functional MRI, one study determined a relationship between amygdala dysfunction and the presence of psychopathic traits based on attentional control [52]. The amygdala not only plays a role in fear processing but also plays a role in attention and detecting the relevance of stimuli [51]. Both psychopathy and narcissism have also been shown to negatively impact decision making, which is oftentimes associated with the ventromedial prefrontal cortex [52]. Brain mechanisms of pathological narcissism and psychopathy demonstrate differences in terms of decision making when compared to healthy controls, which are primarily controlled by the amygdala and vmPFC [51]. Another study conducted by Ueltzhoffer et al. (2023) determined that high-scorers on the Dark Triad showed increased activations in the superior parietal lobule, precuneus, and intraparietal sulcus during decision making [53]. The function of these brain areas in the expression of narcissistic traits should be further explored.

### 7.6. Directions for Future Research

In future research on narcissism and the brain, emphasis should be placed on regions that have been correlated with the characteristics of narcissistic personalities, such as the mPFC, in order to provide more evidence for their involvement in the presence of narcissism. For example, regions of the salience network (including the anterior insula and dorsal anterior cingulate cortex) control responses to negative stimuli such as conflict and pain and have been associated with narcissism [3,47]. Chester and DeWall (2016) found that during high activation of the dorsal anterior cingulate cortex, narcissism was positively correlated with retaliatory aggression after rejection [4]. In another study conducted by Jauk et al., (2017), it was found that the dorsal anterior cingulate cortex was activated during a self-recognition task. The dACC has been associated with social pain seen in individuals with low self-esteem, implying that highly narcissistic individuals may reflect some of these traits [54]. Empathy is additionally controlled via regions of the salience network [47]. Deficits in empathy are seemingly due to improper functioning of the right anterior insula, causing constant default mode network activation and the centering of one’s attention on the self [7]. This self-centered attitude, lack of empathy for others, and need for positive external validation may be linked to an increased appeal to fame and interest in celebrities. Because grandiose narcissism has also been closely linked to an increased desire for fame, the testing of brain regions involved with desire for fame may result in differences in the same areas that have been associated with grandiose narcissism throughout the above studies. The brain regions involved in vulnerable narcissism may eventually be determined to be involved in the occurrence of celebrity worship as well.

## 8. Brain of Celebrity Worshipers

Parasocial relationships (i.e., one-sided relationships) have the ability to greatly impact the lives of celebrity worshipers in terms of their mental and physical health, but not much is known about the neuroscience behind these phenomena. Extensive research has not been performed on the brain regions involved in celebrity worship, the development of parasocial relationships, or the desire to be famous. It is known that narcissism is greatly influenced by the desire to be famous, so it is possible that the brain regions involved with narcissism also impact appeal to fame; however, there is limited information on the brain regions directly related to fame and celebrity worship. 

One study attempted to document the brain activities of both fans and non-fans by employing visually evoked event related potentials (ERPs) to examine people’s attitude toward celebrities when they were presented with a set of photos [55]. The results of the study revealed that the N2 and P300 components seemed to be correlated with differences in reactions between the fan group and non-fan group. In terms of the N2, a larger response was recorded for the fan group when being shown pictures of familiar and unfamiliar faces in comparison to being shown the faces of celebrities. In the non-fan group, their N2 component was similar when being shown all three groups of pictures. Since N2 is associated with cognitive control, the researchers concluded that this difference in reaction might reflect the inhibition of action to familiar and unfamiliar photos due to fans’ anticipation of their favorite celebrity’s photo [55], meaning that they did not react to the familiar and unfamiliar faces because they were specifically waiting for the face of their favorite celebrity to appear. When discussing variations between both groups in P300 amplitudes, the fan group experienced higher amplitudes in response to celebrity photos, while the non-fan group did not experience higher P300 amplitudes. It has been suggested that temporal–parietal P300 components could be related to attentional allocation, which would indicate that fans paid more attention to the photos of their favorite celebrities due to the strong admiration they have for them. The study also provided neurological evidence that “the attitude of fans toward their favorite celebrity was similar to that for their loved child, romantic partner, or other familiar loved ones”, even though the participants and celebrity had never met. Due to the lack of extensive electrode placement or post hoc modeling, specific brain regions were not identified in this study.

Another study compared the performance of a well-learned task (use of dominant hand) to a novel task (use of non-dominant hand) in a word copying exercise when displaying pictures of participants’ favorite television characters [56]. It was found that their performance during exposure to a favorite character was “facilitated on the well-learned task as compared to a control character and inhibited on the novel task as compared to a control character” [56]. It was concluded that the favored television characters were perceived as “more real” in comparison to non-favorites [56], as images of the favored characters may have been more distracting to participants due to a stronger bond or parasocial relationship between the participant and character, triggering social facilitation effects. This result demonstrates how relationships with celebrities have the ability to alter the underlying neural circuits of celebrity worshipers, ultimately affecting their thought processes and cognitive abilities when performing basic tasks. 

In the future, it is necessary to recreate studies such as those listed above and begin research on the neuroscience behind the desire for fame and celebrity worship. Researchers have focused on the neural correlates of being famous, but thus far, they have neglected the impact that celebrities have on their followers and the possible effects on the brains of those followers. It is critical that this type of research starts to be conducted because celebrity worship has a tendency to negatively impact aspects of both mental and physical health [22].

## 9. Conclusions

On a behavioral level, narcissism (a personality type consisting of grandiosity, self-love, and inflated self-views [1]), the desire to become famous, and celebrity worship (an intense form of celebrity attachment [21]) have been linked together in numerous studies [16,17,18,19]. The neural correlates of narcissism have been explored, and frontal regions of the brain such as the medial prefrontal cortex [5,6,10,12] and right dorsolateral prefrontal cortex [5,8,9,12] have been determined to play a pivotal role in the occurrence of narcissistic personality traits. Brain studies on desire for fame and celebrity worship are in their infancy, yet characteristics of these phenomena, such as fantasy proneness [17,36,41], lack of empathy [6,10,47], and unstable self-esteem [11,42,43], point to an overall deficit in self-awareness, which has been connected to the mPFC [37] as well. Overall, the behavioral link between narcissism, desire for fame, and celebrity worship may also indicate that the neural correlates amongst these phenomena are similar—most likely in frontal regions of the brain, such as the mPFC and right dlPFC.

While the evidence is scant, we believe research in this area could assist in elucidating the interplay between self-awareness and lateralized frontal regions as well as default mode circuits. Celebrity worship and desire to be famous are likely unique to our species and reflect some of the more interesting byproducts of our higher-order consciousness. Likewise, narcissism at the clinical and sub-clinical levels is a variable that is critical to celebrity worship, fame, and self-awareness, therefore providing a window into the unique neural architecture of humans. This review, which is the first of its kind, has drawn attention to the increasing utility of examining these variables, and we look forward to the findings and revelations that are to come.
